# Impact of Dietary Regime and Seasonality on Hindgut’s Mycobiota Diversity in Dairy Cows

**DOI:** 10.3390/microorganisms12010084

**Published:** 2023-12-31

**Authors:** Ali Sadek, Bernard Taminiau, Georges Daube, Panagiotis Sapountzis, Frédérique Chaucheyras-Durand, Mathieu Castex, Françoise Coucheney, Djamel Drider

**Affiliations:** 1 Unité Mixte de Recherche (UMR) Transfrontalière BioEcoAgro 1158, Univ. Lille, INRAE, Univ. Liège, UPJV, YNCREA, Univ. Artois, Univ. Littoral Côte D’Opale, ICV—Institut Charles Viollette, 59000 Lille, Francebernard.taminiau@uliege.be (B.T.); georges.daube@ulg.ac.be (G.D.); 2 Lallemand SAS, 19 Rue des Briquetiers, 31702 Blagnac, France; 3 Fundamental and Applied Research for Animal & Health (FARAH), Veterinary Medicine Faculty, Department of Food Sciences, University of Liège, 4000 Liège, Belgium; 4 Université Clermont Auvergne, INRAE, UMR 0454 MEDIS, 63000 Clermont-Ferrand, France; panagiotis.sapountzis@inrae.fr

**Keywords:** dairy cows, mycobiota, metataxonomic analysis, *Geotrichum*

## Abstract

We describe and discuss the intestinal mycobiota of dairy cows reared in France following variations in dietary regimes and two seasons. Two groups of 21 animals were followed over a summer and winter period, and another group of 28 animals was followed only during the same summer season. The summer diet was based on grazing supplemented with 3–5 kg/d of maize, grass silage and hay, while the winter diet consisted of 30% maize silage, 25% grass silage, 15% hay and 30% concentrate. A total of 69 DNA samples were extracted from the feces of these cows. Amplification and sequencing of the ITS2 region were used to assess mycobiota diversity. Analyses of alpha and beta diversity were performed and compared statistically. The mycobiota changed significantly from summer to winter conditions with a decrease in its diversity, richness and evenness parameters, while beta diversity analysis showed different mycobiota profiles. Of note, the *Geotrichum* operational taxonomic unit (OTU) was prevalent in the winter group, with a mean relative abundance (RA) of 65% of the total mycobiota. This *Geotrichum* OTU was also found in the summer group, but to a lesser extent (5%). In conclusion, a summer grazing diet allowed a higher fecal fungal diversity. These data show, for the first time, that a change in diet associated with seasonality plays a central role in shaping hindgut fungal diversity.

## 1. Introduction

Ruminants contribute significantly to global food security by providing adequate amounts of protein and energy to humans [1]. The rumen, the largest digestive compartment in the ruminant gastrointestinal tract (GIT), harbors a complex consortium of bacteria, archaea, fungi, viruses and ciliated protozoa that interacts to degrade feed and provide metabolic by-products and nutrients to the host [2]. The microbial community produces organic acids such as acetic acid, propionic acid and butyric acid, which provide 70% of the energy requirements of the host organism [3,4]. Most of the digestion takes place in the rumen, but the remaining part of the digestive process can take place in the large intestine, where resident microbes break down dietary compounds that have not been digested or absorbed in the upper parts of the GIT [5,6]. These dietary compounds and associated microbiota can affect gut integrity, which is paramount to maintaining animal health, performance and well-being due to local and systemic inflammation that occurs with infiltration of luminal contents across the epithelium [7,8].

An adequate intestinal barrier is necessary to prevent the entry and diffusion of ruminal pathogens, which could produce a number of virulence factors and then evade the host defense mechanism [9]. Therefore, a better knowledge and control of the intestinal microbiota of ruminants is particularly relevant in view of its significant contribution to the overall digestion and maintenance of intestinal health. A number of studies have already assessed ruminal microbial diversity using culture-dependent methods [10], which have allowed the isolation and cultivation of ~15% of the global rumen bacterial population [10]. 

The last decade has seen a breakthrough in next-generation sequencing (NGS), which provides rapid, reproducible and comprehensive tools for qualitative and quantitative assessment of rumen microbial diversity [10,11,12,13]. Bacteria constitute the largest population with ~10^12^ cells/gram [14,15], and a diversity of at least 7000 species distributed in 19 phyla, dominated by *Firmicutes* (56%), *Bacteroidetes* (31%) and *Proteobacteria* (4%) [16]. Ciliated protozoa in the rumen play a role in volatile fatty acid (VFA) production and, together with archaea, are major producers of methane, resulting in a net energy loss [17]. The abundance of archaea in the rumen varies from 108 to 1010 gene copies per gram [18] and is dominated by the genera *Methanobrevibacter* (>60%) and *Methanomicrobium* (15%). [16]. Ciliated protozoa are thought to stabilize rumen pH when animals are fed diets high in available starch, and their abundance is estimated to be between 10^5^ and 10^6^ cells per gram of rumen content, with *Entodinium* being the most dominant genus [14].

Rumen anaerobic fungi (AF) were initially mistakenly thought to be protozoa because of their zoospore with flagella [19,20]. Orpin [21] correctly classified them decades later [21]. These fungi are endowed with several enzymes involved in fiber degradation [22], and their abundance has been reported to be ~10% of the total microbial biomass [23]. They are known to be the first to locate, adhere to and colonize plant biomass in the rumen [24,25]. Later, rumen AF were included in the *Neocallimastigomycota* phylum, which consists of a unique order, *Neocallimastigales*, and a unique family, *Neocallimastigaceae* [26]. While the ruminant digestive tract bacteriome has received much attention, studies aimed at characterizing its fungal content and diversity have started to emerge. In light of this, Koester et al. [27] compared fecal bacterial and fungal communities in Angus cows exposed to the endophytic fungus *Epichloe coenophiala*, which is responsible for fescue toxicosis (FT). Consequently, animals showed contrasting tolerance to FT. For example, groups with high tolerance to FT exhibited more diverse fecal microbial communities, with a high abundance of AF belonging to the *Neocallimastigomycota* phylum, which is known for its cellolytic activities. However, groups with low tolerance to FT had a higher abundance of phylotypes within the genus *Thelebolus*. 

The presence of AF such as *Pecoramyces*, known for their elevated lignocellulolytic activities, has been reported in cattle and sheep feces [28]. In a recent study, Meili et al. [29] also found that host phylogeny had a greater impact on the mycobiome than domestication status or biogeography when examining 6661 fecal samples. Fungal communities found in fecal samples can be considered a good representation of the gut community, as feces can serve as an artificial proxy for the gut compartment [30,31]. 

As a global effort to describe the ruminant GIT mycobiota, we provide here a set of data highlighting the dietary regime shift associated with seasonality on cow fecal mycobiota. The diets consisted of outdoor grazing in summer and a controlled diet in winter in an experimental farm in France. It should be noted that the mycobiota diversity evaluated in this study was performed on the same DNA samples extracted and used by Teseo et al. [31], who evaluated the fecal bacteriome and its putative functions in the feces of these animals. 

## 2. Materials and Methods

### 2.1. Animal Rearing and Sample Collection

The collection of fecal samples was previously reported by Teseo et al. [31]. Briefly, samples were collected from September to December 2020 from lactating Holstein dairy cows kept at the INRAE Herbipole experimental unit (UE 1414; Saint-Genes-Champanelle, France). When fed the summer diet in September, the cows grazed on a permanent pasture close to the farm and were supplemented with a ration of maize and grass silage and hay. When the winter diet was fed in December, the cows had ad libitum access to a standard diet consisting of 30% maize silage, 25% grass silage, 15% hay and 30% concentrate. Out of 50 cows, fecal samples were collected from 28 animals in September only and from 21 animals (paired cows) in both September and December, resulting in a total of 70 fecal samples. Samples were stored at −80 °C prior to DNA extraction. 

### 2.2. DNA Extraction

DNA extraction was performed as described by Teseo et al. [31]. Briefly, the Quick-DNA Fecal/ Soil Microbe Miniprep Kit (D6010, ZymoResearch, Tustin, CA, USA) was used to extract total DNA from 250 mg feces according to the manufacturer’s instructions. The Nanodrop spectrophotometer (Nanodrop 1000, Thermo Fisher Scientific, Inc., Waltham, MA, USA) was used to determine the quality and purity of the extracted DNA. 

### 2.3. Amplicon Sequencing and Processing

Fungal profiling targeting the internal transcribed spacer 2 (ITS2) region was performed as previously described [32] using Illumina MiSeq technology (Illumina, SY—410-1003). Briefly, sequencing libraries were prepared using the ITS3KYO2 forward primer (5′-GATGAAGAACGYAGYRAA-3′) and the ITS4 reverse primer (5′-TCCTCCGCTTATTGATATGC-3′) with Illumina overhand adapters. PCR products were purified using the Agencourt AMPure XP Beads Kit (Beckman Coulter, Pasadena, CA, USA). Indexing PCR was performed using Illumina Nextera XT index primers (1 and 2). Raw amplicon sequencing libraries were submitted to the NCBI database under the bio-project number ID PRJNA942252. Sequence read processing was performed using the MOTHUR software package v1.47 for sequence cleaning, taxonomic assignment and OTU clustering (with the commonly used threshold of 0.03 distance cut-off) [33,34], and the VSEARCH algorithm for chimera detection [35]. ITS2 reference alignment and taxonomic assignment were based on the UNITE database v9.0, which is considered the gold standard dataset in fungal ecology studies for its systematic curation and error filtering of ITS sequences [36]. OTUs that were not assigned to the genus level were manually selected and searched on the BLAST server hosted at the National Center for Biotechnology Information (NCBI, http://www.ncbi.nlm.nih.gov/BLAST/ (accessed on 27 July 2022)) [37]. Only one more OTU was identified taxonomically using this method. The OTU name was changed from the family *Dipodascaceae* to the genus *Geotrichum*.

### 2.4. Diversity and Statistical Analyses 

Alpha diversity parameters for fungal diversity (inverse Simpson’s index), richness (Chao1 index) and evenness (Simpson’s index-based measure) and Good’s coverage index were calculated using MOTHUR v 1.47. 

Beta diversity was visualized with a Bray–Curtis dissimilarity matrix-based nonparametric dimensional scaling (NMDS) model using the vegan (https://cran.r-project.org/web/packages/vegan/index.html; accessed 14 June 2023) and vegan3d (https://cran.r-project.org/web/packages/vegan3d/index.html; accessed 14 June 2023) packages in R.

Statistical differences in alpha diversity indices between the two diet groups were assessed using PRISM 9.0 with either the Mann–Whitney test or the Wilcoxon matched-pairs signed rank test for unpaired and paired animals. Beta-diversity differences between diets were assessed with an analysis of molecular variance (AMOVA) [38] and a homogeneity of molecular variance (HOMOVA) beta-dispersion analysis [39] using MOTHUR v1.47. Differences in alpha and beta diversity were considered significant if the *p*-value was less than 0.05. Differential fungal population abundance analysis between diets was performed with the DESEQ2 package, with a Benjamini–Hochberg false discovery rate procedure for multi-test correction (Q-value: 0.05) using R [40].

## 3. Results

Of the total DNA extracted, 69 of the 70 samples were used. In fact, one sample from 29 additional cows from the summer period could not be sequenced on the Illumina MiSeq platform and was removed from the rest of the study. Starting with 8,238,877 raw reads (median length 269 nucleotides), we obtained 7,548,833 reads after cleaning and chimera removal. A rarefaction table with 10,000 reads per sample was used for OTU clustering (16,851 OTUs in total). The OTUs were clustered into phylotypes of 487 genera. Finally, only phylotypes belonging to the kingdom Fungi (414 phylotypes in total) were retained for further diversity analysis. A mean sampling Good’s Coverage of 99.75% was obtained, indicating a high degree of coverage by the Miseq sequencing unit. 

### 3.1. Alpha Diversity Analysis Revealed a Reduction in Mycobiota Richness, Diversity and Evenness from Summer to Winter Conditions

**Figure 1:**
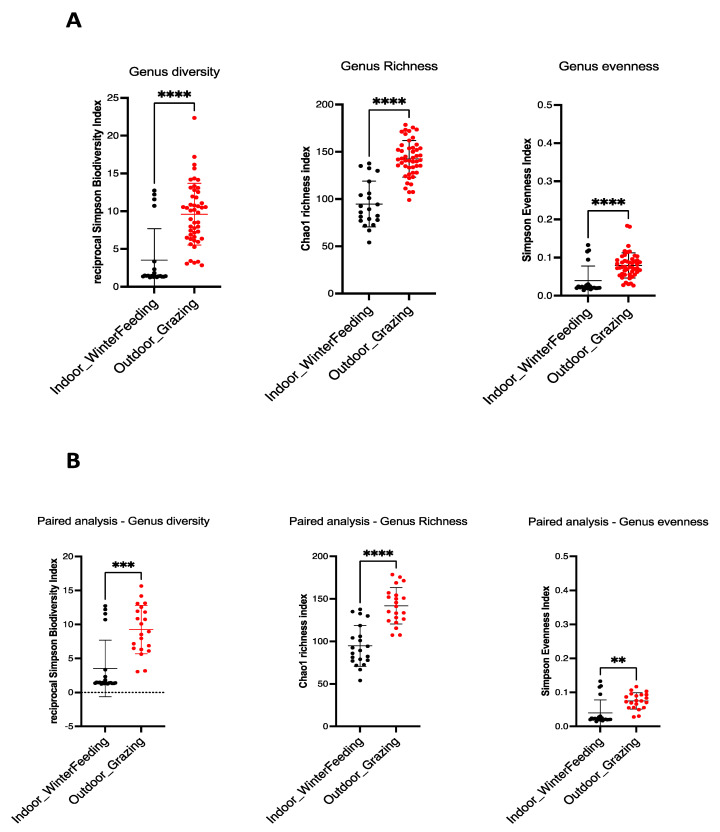
Alpha diversity indices in winter and summer dairy cow dung. Fungal intrinsic diversity was derived from Simpson’s reciprocal diversity index, fungal richness from Chao1 index, and fungal genus evenness from Simpson’s index. For all cows (**A**), a significant statistical decrease was observed from outdoor diet in summer (red dots) to indoor diet in winter (black dots) (unpaired *t*-test for Chao1 index and Mann–Whitney test for Simpson reciprocal biodiversity and Simpson evenness index, *p*-value: <0.0001 (****) for all). For paired cows (**B**), the same observation is made (unpaired *t*-test for Chao1 index and Mann–Whitney test for reciprocal Simpson biodiversity and Simpson evenness index). *p*-value = 0.001 (***)for reciprocal Simpson biodiversity index, *p*-value = 0.0001 (****) for Chao1 richness index and *p*-value = 0.01 (**) for Simpson evenness index.

Alpha diversity is commonly used to assess the diversity within a community, independent of external elements. For this purpose, ecological parameters such as Chao1 index (richness indicator), Simpson evenness (uniformity indicator) and Inverse Simpson (diversity indicator) are used. The ecological indices of the mycobiota of the sampled feces are shown in Figure 1. A statistical decrease in alpha diversity was observed for all animals when shifting from outdoor to indoor feeding, which occurred in summer and winter (48 cows), respectively, for the Chao1 index (unpaired *t*-test, *p*-value: <0.0001), Simpson evenness and Inverse Simpson (Mann–Whitney test, *p*-value: <0.0001) (Figure 1A). The same observation was made for paired cows (B) (*p*-value = 0.001 for Chao1 index and Simpson evenness, *p*-value = 0.0001 for Inverse Simpson) (Figure 1B). These results clearly indicate the decrease in all alpha diversity indices (diversity, richness, evenness) when the diet of dairy cows was changed from outdoor grazing in summer to a more controlled indoor diet in winter.

### 3.2. Beta Diversity Revealed two Distinct Mycobiota Profiles

**Figure 2:**
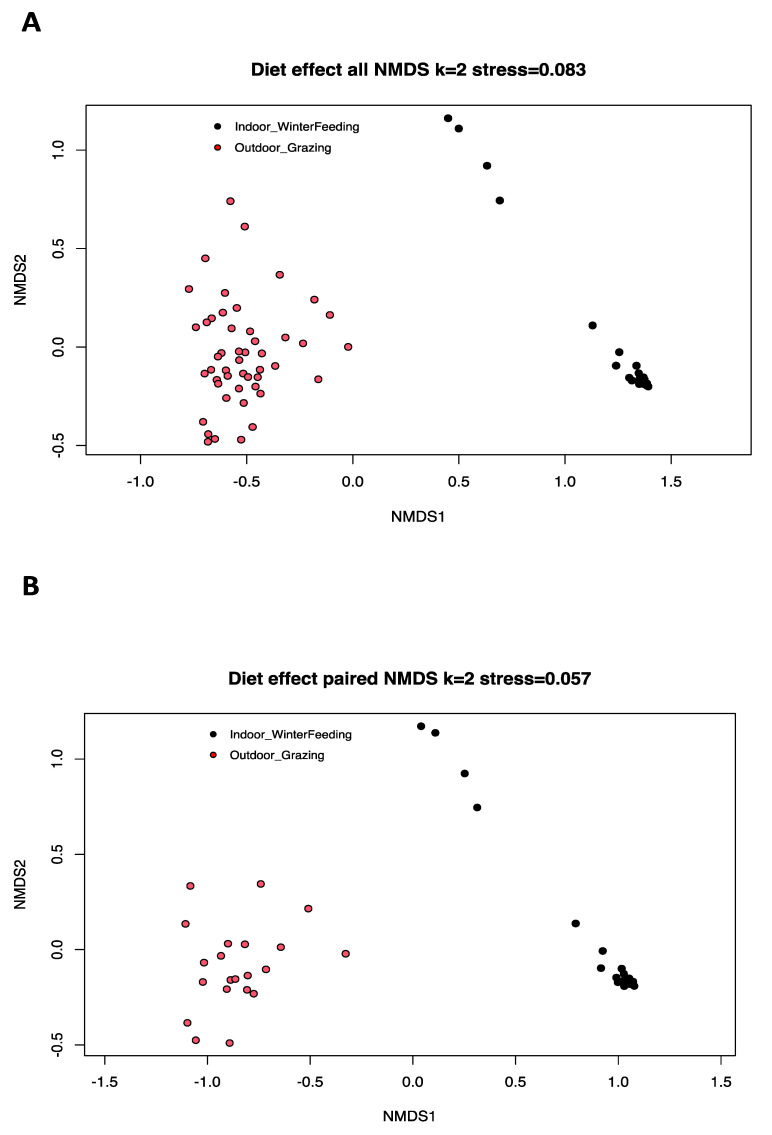
Nonmetric multidimensional scaling (NMDS) ordination based on a Bray–Curtis dissimilarity matrix of fecal mycobiota profiles of all dairy cows used in our study (**A**) and of paired cows (**B**) with two different diets (red = summer pasture grazing, black = winter controlled feed intake). The AMOVA results showed a heterogeneous variance in beta diversity (*p*-value: <1 × 10^−5^) and the HOMOVA test showed no statistical difference (*p*-value = 0.13) for all cows. Differences were observed for AMOVA (*p*-value: <1 × 10^−5^) and HOMOVA (*p*-value = 0.0006) analysis for paired cows.

Beta diversity is the typical way to compare the variation in species composition between different microbiota, and is based here on a Bray–Curtis dissimilarity matrix. Significant differences were found between the two diet categories based on AMOVA analysis (*p*-value: <1 × 10^−5^) (Figure 2A). AMOVA assesses the variance of molecular parameters between two population groups. No significant statistical difference was found for the HOMOVA test (*p*-value = 0.13). HOMOVA evaluates the genetic diversity within the two selected cow populations to determine if there is a significant difference in genetic diversity within each group. For paired cows, differences exist with AMOVA (*p*-value: 0.00001) and HOMOVA (*p*-value = 0.0006), and the beta diversity of the mycobiota was visually represented with a nonmetric multidimensional scaling (NMDS) model (stress < 0.1, two dimensions) in Figure 2B.

### 3.3. Fungal Populations and Description of the Core Mycobiota

**Figure 3:**
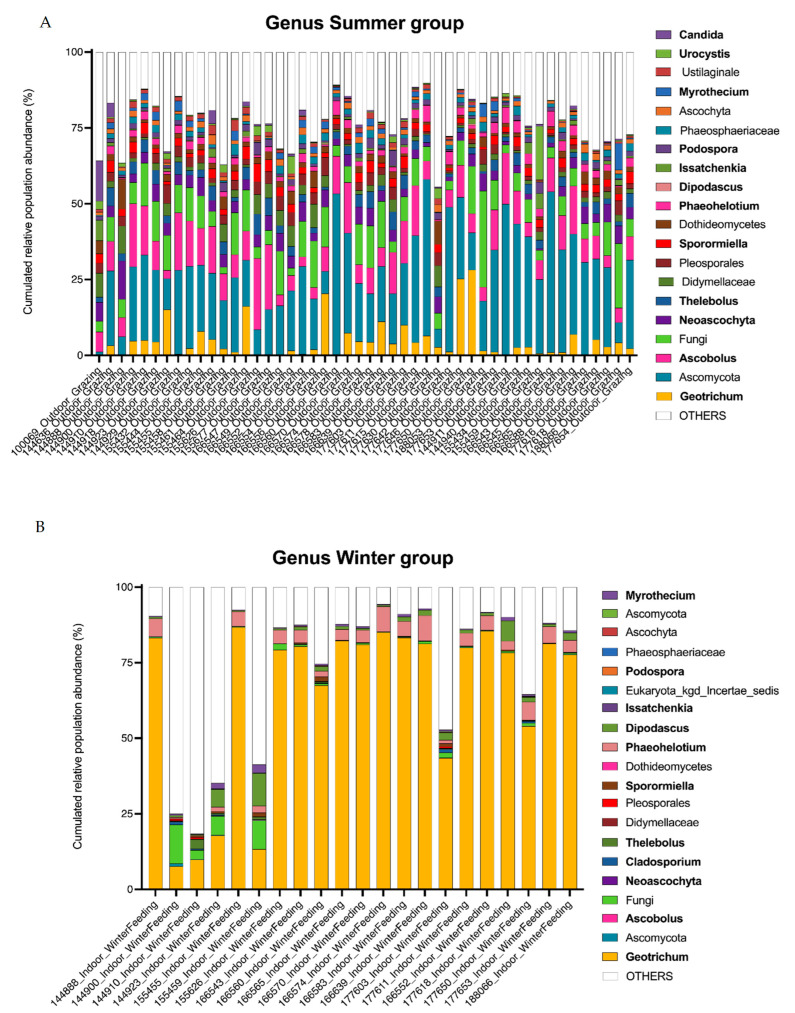
Fungal population in the large intestine of dairy cows by ITS2 profiling (**A,B**). Bar graphs show the relative abundance of the major fungi identified (top 20 OTUs). Identified genera are shown in bold.

A total of 414 operational taxonomic units (OTUs) belonging to the kingdom Fungi were retained for further analysis. Overall, 338 OTUs were identified to the genus level. For each sample obtained from animals receiving one of the two diets, the distribution of the main genera and their relative abundance (RA) in the feces are presented in Figure 3. The most abundant phylum was Ascomycota, with a mean relative abundance (RA) of 84% in summer and 86% in winter, followed by Basidiomycota with a mean RA of 5% in summer and 3% in winter, and *Neocallimastigomycota* with a mean RA of 1% in summer and 6% in winter (Figure 3A). One unidentified phylum (Designated Fungi) was significantly present in the summer diet with a mean RA of 10% and 2% in the winter (Deseq2, log2foldChange = 4.0, *p*-value = 2.66 × 10^−21^). Five OTUs were shared between the summer group samples and constituted the core mycobiota, i.e., OTUs were shared with a relative abundance of >1% in each sample as described by McFarland et al. [41]. These five OTUs were assigned to *Ascomycota*, *Ascobolus*, Fungi, *Thelebolus* and *Sporormiella*. In the winter group, the OTU corresponding to *Geotrichum* was the only one common to all cows (Figure 3B). Among the unicellular fungi present in the 20 most abundant OTUs, those corresponding to the genera *Geotrichum* and *Dipodascus* were found in winter (Deseq2 summer versus winter, log2foldChange = −3.3, *p*-value = 4.17 × 10^−20^ and log2foldChange = −5.0, *p*-value = 1.11 × 10^−30^, respectively). *Issatchenkia* was more abundant in winter (mean RA 2%) compared to summer (mean RA 0.9%), but no statistical difference was found in DeSeq2 analysis. The percentage of relative abundance is provided in Table S1 as supplementary data for most OTUs.

## 4. Discussion

The rumen microbiota of ruminants is relied upon to promote health and well-being through the digestion of feed components such as cellulose, hemicellulose, starch, proteins, lipids and their conversion to volatile fatty acids (VFAs) and microbial proteins, which are then absorbed. Furthermore, microbial diversity is closely related to metabolic activities [42] and could be influenced by various factors such as animal age, environment, seasonality and diet. It has been previously reported that ruminal microbiota could be modified by dietary and additive supplementation [43]. In order to gain insight, it seemed advantageous to assess other compartments and determine the microbial community content of the entire GIT. The bacterial part of the microbiota has been extensively studied in dairy cows for many years [44,45,46,47]. However, fungi, and in particular the yeast population, have been less studied than their bacterial counterparts. Thus, several studies have aimed at describing the yeast communities present in the rumen [48], evaluating changes according to the age of the animals [49] and different roughage/concentrate ratios [50] or after an additive treatment [51]. Other studies aimed to investigate the potential enzymatic activity [52] or to isolate potential new probiotics [53,54,55].

Our study was dedicated to the mycobiota composition and diversity of cow feces and its potential modification due to different diets and environmental factors such as summer and winter constraints. This snapshot of the mycobiota was obtained by amplicon sequencing of fecal DNA from 50 dairy cows, 21 of which were subjected to a diet change from summer to winter. Amplicon-based analysis is necessary when studying the fungal community due to the relatively low abundance of eukaryotic DNA compared to prokaryotic DNA. In this regard, Teseo et al. [31] found in the same metagenomically analyzed samples that more than 61% of the predicted open reading frames were of bacterial origin, while 0.14% were from Eucarya. Of note, 38% of the reads in this analysis were unclassified [31]. Another study by Meili et al. [29], also failed to use the metagenomic shotgun sequencing technique and linked this drawback to the low amount of anaerobic gut fungal DNA in their samples compared to bacterial DNA; a hypothesis confirmed by using quantitative PCR assay. Also, the lack of publicly available genomes, especially those from AF, makes functional studies of fungi uncertain. The first available sequenced genome of an AF was obtained from *Orpinomyces* in 2013 [56]. Targeted metagenomics is still considered the most appropriate method for mycobiota community analysis. Regarding feces, the fecal microbiota has the advantage of being easily accessible and could serve as a non-invasive approach to decipher the hindgut microbiota [30,44,57].

In the present study, we obtained 414 OTUs belonging to the kingdom Fungi, but identification of only 76 of these OTUs was achieved at the genus level. Interestingly, we identified OTUs belonging to the family *Neocallimastigaceae*, which are commonly found in the rumen. They often play a role in fiber degradation with their ability to colonize and penetrate plant tissues, their wide range of active enzymes such as cellulase and their ability to work in synergy with ruminal bacteria [58,59,60,61]. In this context, genes encoding these enzymes are being cloned for heterologous production in biotechnology for cellulose, hemi-cellulose and lignin degradation, and a recent and updated list has been published by Hooker et al. [62]. In agreement with our study, AF have also been found in feces by other authors [29,63,64]. Next, our data analysis showed that at the phylum level, *Ascomycota* was found in 84% and 86% of the RA in the summer and winter groups, respectively, with a clear difference in diversity in terms of OTUs. These data are consistent with those reported by Zaman et al. [65] and Ji et al. [55]. The datasets from our study underline the strong dependence of the hindgut mycobiota on the diet, with increased alpha diversity observed with grassland grazing. Fungal content was also examined on paired cows and was statistically tested using the HOMOVA test, which showed a clear statistical difference (Bray–Curtis dissimilarity, *p*-value = 0.0006).

Our results indicate a significant decrease in alpha diversity when the diet of dairy cows was changed from outdoor grazing in summer to a more controlled indoor diet in winter. These results mirror those reported by Teseo et al. [31] when assessing the bacterial population in the same fecal samples. Teseo et al.’s functional metagenomic study also highlighted the fact that higher diversity taxa of bacteria carry more enzymes associated with the uptake of monosaccharides, resulting in the production of short-chain fatty acids (SCFAs) such as acetate, propionate and butyrate pathway related enzymes, which are more abundant in summer than winter [31]. The functional fungal study could help answer whether the most diverse mycobiota found with the summer diet could be correlated with better digestion of the remaining feed intake in the gut, like the role played by the bacterial microbiota.

We also identified unicellular fungi such as *Geotrichum* and *Issatchenkia*. Next, *Geotrichum candidum* (teleomorph = *Galactomyces candidus*) was initially classified as a yeast, belonging to the family *Dipodascaceae*, a subdivision of *Saccharomycotina* from the phylum *Ascomycota* within the kingdom Fungi [66]. This species has been reclassified as a filamentous yeast-like fungus due to its morphological and phenotypic characteristics, which are close to those of fungi. Remarkably, this saprophytic yeast-like fungus has been found in the GIT of animals, including cattle, and has been associated with cases of bovine geotrichosis. [67]. *G. candidum* has also been suggested as a probiotic. An in vitro study reported the production of anti-*Listeria* extracellular compounds [68] and phenyllactic acid capable of inhibiting *Fusarium sporotrichoides* and *F. langsethiae*, mycotoxin-producing fungi [69]. In ruminants, it has been applied to production for its ability to improve feed efficiency, milk yield, growth performance and anaerobic bacteria counts, as well as reduce pathogen counts in 12 experimental dairy cows [65]. Notably, *Geotrichum* is not only the main core of the fecal mycobiome in winter, but also the most abundant OTU (mean RA of 65%). In some samples, it represents up to 87%, emphasizing its predominance in the colonic mycobiota. With such a high abundance, it should be easily isolated from winter fecal samples on fungal selective media (such as Sabouraud agar plate supplemented with chloramphenicol) to assess probiotic properties in order to better understand its role in the gut. As for *Issatchenkia*, this yeast is within the kingdom Fungi, and is the same phylum and subdivision as *Geotrichum*, but belongs to the family *Saccharomycetidae*. While its role in the global mycobiota remains unknown, the species *Issatchenkia orientalis* has been tested in vitro as a potential microbial feed additive for ruminants [70]. According to Rodriguez et al. [71], *I. orientalis* stimulated in vitro gas production of Tifton hay, while other studies reported that the addition of high levels of *I. orientalis* resulted in a reduction in fiber digestibility in ruminal fermentation in vitro [72]. In addition, *I. orientalis* has been shown to reduce the adherence of a pathogenic *Candida albicans* in vitro [73]. Little information is available about the endogenous yeast population, although several studies have reported the beneficial effects of exogenous yeast probiotics supplemented through the diet [74,75,76]. No core mycobiota was found among all animals on the two different diets, but common OTUs within the summer group were identified as *Ascobolus*, *Thelebolus*, *Sporormiella*, *Ascomycota*, Fungi. For the three identified genera, it is not uncommon to find them in the feces of the animals, especially *Thelebolus*, which has already been found in ruminant [77]. In a previous study, this genus was associated with the production of a bioactive exopolysaccharide, thelebolan, with anti-inflammatory properties [78], supporting its use as a host health promoter. Unfortunately, the fungi and *Ascomycota* OTUs could not be identified more precisely. A number of questions remain to be answered as to whether these fungi are naturally present in the gut or are primarily promoted by the animal’s diet. Similarly, it remains to be determined whether they are natural inhabitants based on summer or winter feed intake, or if they are introduced through the animal’s diet. Our study did not focus on the fungal population of the diet, but this mycobiota analysis could help to answer these open questions. 

In a constant effort to provide more taxonomic precision on the mycobiota of the ruminant’s sample, we suggest the use of other technologies based on the sequencing of longer fragments. Historically, AF have been identified taxonomically by the sequence of the internal transcribed spacer 1 (ITS 1) region, although this marker has limitations such as a high variation of clones from a single culture isolate (up to 13%) [79]. The use of the ITS region is common and widely used in fungal and yeast population studies [34,80], and the comparison between ITS1 and ITS2 region as selected regions for fungal identification showed similar results [81]. While this region is still a reference and is likely to be used in the future for ecological studies of the fungal microbiota, more advanced taxonomic resolution and a more curated database are needed. Thus, the third-generation sequencing technology proposed by PacBio or Oxford Nanopore seems to provide more informative data, allowing identification at the genus or species level. Sequencing a targeted longer fragment, such as the entire ITS region to the end of the large 28S subunit, might be appropriate. In fact, it was performed on pure culture by Wurzbacher et al. [82] on *Chytridiomycota*, *Basidiomycota* and the rare *Nephridiophagidae*. In their amplicon-based work, Meili et al. [29] found 56 novel genera with Illumina sequencing data. However, when the same samples were compared with the Pacbio-generated output, 49 of these 56 new genera were finally identified. In addition to these new sequencers, efforts are needed to provide a complete and curated database in the future to develop the long-read approach for fungi [22]. In our study, 81.6% of the OTUs, representing 338 OTUs, were assigned to the genus level, and next-generation sequencing platforms could be helpful for the remaining 18.4% or to more precisely identify the mycobiota population within our samples at the species level. 

## 5. Conclusions

This study allowed us to provide snapshots of the hindgut mycobiota of dairy cows and contributes to global advances in understanding ruminal fungal diversity and its modulation by diet and environmental factors of summer and winter seasons. A pasture diet resulted in greater fungal richness and diversity in the cow’s hindgut. Of note, the change in diet from summer to winter decreased fungal diversity and richness. The observed shift in the hindgut fungal population occurred over a relatively short period of time. Note that no common core mycobiota to both seasons was found, and *Geotrichum* prevailed in winter rations. To our knowledge, this is the first report of such a significant relative abundance of *G. candidum* in the mycobiota of a ruminant. Further analyses are required to understand the role of *Geotrichum*, but also that of other unicellular or multicellular fungi inhabiting the ruminal hindgut, leading to the identification of potential new probiotics for dairy cows. The use of long-read sequencing technologies for a more precise taxonomic assignment at the species level is the obvious next step for dairy cow fecal mycobiota analysis. 

## Data Availability

Data of this study are available in the NCBI database under the bio-project number ID PRJNA942252, and in the Table S1.

## Supplementary Materials

The following supporting information can be downloaded at: https://www.mdpi.com/article/10.3390/microorganisms12010084/s1, Table S1: Percentage of relative abundance for most OTUs in this study.
